# Evaluation of the autonomic nervous system by analysis of heart rate variability in the preterm infants

**DOI:** 10.1186/s12872-019-1166-4

**Published:** 2019-08-16

**Authors:** Luiz Fernando Martins de Souza Filho, Jordana Campos Martins de Oliveira, Mayara Kelly Alves Ribeiro, Marcelo Cozac Moura, Nelson David Fernandes, Rafael Dias de Sousa, Gustavo Rodrigues Pedrino, Ana Cristina Silva Rebelo

**Affiliations:** Universidade Federal de Goiás, Avenue Esperança, Chácaras de Recreio Samambaia, Goiânia, Goiás 74690-900 Brazil

**Keywords:** Preterm infants, Heart rate, Autonomic nervous system, Heart rate variability, Postnatal adaptation, Cardiac autonomic nervous system

## Abstract

**Background:**

Premature infants may present with damage to the autonomic nervous system (ANS), which may be related to poorer neurological development. Among the techniques used to evaluate the ANS, heart rate variability (HRV) emerged as a simple, non-invasive, and easy to apply tool. The aim of the present study was to analyze and compare HRV in preterm infants at different times of hospitalization in order to verify the possible environmental relationships or clinical evolution with HRV.

**Methods:**

A longitudinal, prospective, and descriptive study with non-probabilistic sampling composed of 25 collections of preterm infants of HRV at two moments: moment I (within 15 days of birth) and moment II (after 45 days post-birth). The Polar V800 heart rate monitor was used with the Polar H10 cardiac transducer to collect HRV, which was collected in the supine position for 15 min. The HRV data were analyzed by the linear method in frequency domain and time domain and by the nonlinear method using Kubios HRV analysis software, version 3.0.2.

**Results:**

There was an increase in HRV values at moment II, these being statistically significant in the SD1, ApEn, and SampEn. Data related to increased sympathetic nervous system activity, parasympathetic nervous system activity, and increased index complexity.

**Conclusions:**

The data demonstrate an increase in HRV values in premature infants at moment II, demonstrating a possible development in the maturation of the ANS during hospitalization. Trial registration: RBR-3x7gz8 retrospectively registered.

**Electronic supplementary material:**

The online version of this article (10.1186/s12872-019-1166-4) contains supplementary material, which is available to authorized users.

## Background

Preterm birth is unpredictable and affects all social classes. It occurs at a higher rate in poorer mothers, generating social and financial costs that are difficult to measure, and it is one of the main determinants of the risk of dying in the neonatal period [1–3]. It is estimated that 3.6 million deaths worldwide occur annually in the neonatal period, and those related to prematurity account for 29% of these deaths [4].

Premature infants may have impairments in the autonomic nervous system (ANS). This system plays an important role in the regulation of the physiological processes of the organism, both in normal and pathological conditions, through the balancing action of the sympathetic nervous system (SNS). The parasympathetic nervous system (PNS) branches of the ANS control the heart rate (HR). In preterm infants, changes in the ANS may suggest less neurological development [5, 6].

Among the techniques used to evaluate the ANS, heart rate variability (HRV) has emerged as a simple, non-invasive, and easily applicable tool. Using this method, it is possible to verify the oscillations in the interval between consecutive RR intervals and between consecutive instant HR [6, 7]. Changes in HRV patterns provide a sensitive and anticipatory indicator of health impairment. Lower HRV values indicate abnormal adaptation with ANS insufficiency, while high values ​​indicate good adaptation and efficient autonomic mechanisms [6, 8].

In preterm infants, the behaviour of HRV is less complex compared to that in full-term neonates, and this relationship is suggestive of less neurological development in preterm infants [5, 6]. The inclusion of HRV analysis as a supplementary tool becomes relevant in preterm infants to verify maturation autonomy and progression to eutrophy as an action directed to the development and strengthening of health services, where this evaluation will allow data previously distant from clinical reality to be covered [9, 10]. However, the literature remains restricted in relation to studies that evaluate the HRV of preterm infants, mainly related to its clinical evolution.

In this sense, the aim of this study was to analyse and compare the HRV in preterm infants during two periods of hospitalization, namely, within 15 days of birth and after 45 days post-birth, to verify the development of a preterm infant sample during hospitalization.

## Methods

### Design

This was a prospective, descriptive, longitudinal observational study.

### Ethical aspects

The study was conducted in accordance with the directives and norms regulating research involving human beings (Resolution 466/2012 of the National Health Council), submitted and approved by the Research Ethics Committee of the Universidade Federal de Goiás (opinion no. 636.368). This study was derived from the Cardiac Autonomic Modulation, Anxiety and Depression in Mothers of Preterms Submitted to Music Therapy Intervention: Randomized Controlled Trial, registered in the Brazilian Registry of Clinical Trials (register number RBR-3x7gz8).

To participate in the study, a formal invitation was made to the guardians and/or the parents of the preterm infants, who were invited to read and sign the informed consent form, being advised of their ability to withdraw consent at any time without any encumbrance. Prior to the performance of each of the evaluations of HRV, a request was made for formal written authorization from the multidisciplinary team of the service and parents and/or guardians.

### Sample

A non-probabilistic sample comprised of preterm infants (30.52 ± 2.86 weeks) at a mean birth weight of 1.52 ± 0.65 kg at admission to the Hospital e Maternidade Dona Íris was evaluated for eligibility to participate in the study. Clinically stable preterm infants of both sexes were included.

Initially, 40 preterm infants were chosen upon hospital admission. Twenty-five preterm infants were ultimately selected, making it possible to perform 50 collections at two moments: moment I (within 15 days of birth) and moment II (after 45 days post-birth). The preterm infants who became agitated during HRV collection were excluded.

### Procedure

The evaluation of HRV was performed in the hospital bed of each preterm infant by a previously trained researcher. For the evaluation of HRV, a validated portable device, Polar V800, was used, which stands out for producing recordings of consistent RR intervals comparable to the electrocardiogram (ECG), and like the other portable HRV capture devices have been widely used for presenting lower cost, small amount of absolute error in relation to the ECG and allow an improvement in the practicality and conformity of the collection as well as parameters of HRV highly comparable [7, 11, 12].

In conjunction with the Polar V800 heart rate monitor, an adaptation of the transmitter was used the Polar H10 cardiac transducer to capture the RR intervals, placed in the chest region in the 5th intercostal space and later transferred through an interface to a compatible computer.

The recording of HR and RR intervals for evaluation of the cardiovascular autonomic control was performed under resting conditions in the supine position for 15 min.

### Heart rate variability analysis

For the HRV analysis, stretches of greater signal stability with at least 256 consecutive beats were selected [13]. The analysis was performed using linear and non-linear models.

For linear HRV analysis in the time domain, the rMSSD (square root mean square differences between adjacent normal RR intervals in one time interval), SDNN (standard deviation of all normal RR intervals), and pNN50 (percentage of adjacent RR intervals with a duration difference greater than 50 ms) indices were included. The rMSSD and pNN50 indices reflect the PNS, and the SDNN reflects the SNS and PNS [6].

For non-linear analysis of HRV using geometric methods, the indices obtained from the Poincaré plane, SD1 (dispersion of points perpendicular to the line of identity that appears to be an index of instantaneous recording of beat-to-beat variability), SD2 (points scattered along the identity line, which represent the HRV in a long-term record), and SD1/SD2 (ratio between short and long variances of RR intervals) express the complexity of HRV. The SD1 index reflects the PNS, and the SD2 and SD1/SD2 ratio reflect the SNS and PNS [6].

The entropy analysis was performed by approximate entropy (ApEn), which represents the randomness or predictability of physical systems. ApEn measures the degree of irregularity and complexity of the signal, with the degree of complexity being proportional to the presented value; the higher the value is, the greater the RR intervals. Another method of analysing the entropy used was the sample entropy (SampEn), a measure similar to ApEn, but it expresses the disorder presented in the series, where higher values are associated with healthy individuals and smaller values are associated with heart failure [13, 14].

The HRV data were analysed by the linear time domain method (RR intervals, SDNN, rMSSD, and pNN50) and by a non-linear method (SD1, SD2, SD1/SD2, ApEn, and SampEn) by Kubios HRV analysis software, version 3.0.2.

### Statistical analysis

For the initial comparison of groups, data normality was determined (Shapiro–Wilk test), and when the normal distribution was accepted, Student’s t-test for unpaired data was applied. In situations where the normal distribution was not accepted, the Mann–Whitney test was applied. Differences in these tests were considered statistically significant when the *p*-value was less than 0.05.

The correlation between the data obtained in the HRV and the age of the preterm infants at the moment of the evaluation was verified using the Pearson test when the data were considered to have a normal distribution. When normality was not accepted, the Spearman test was applied, and reference data with 0.1 to 0.3 as a low correlation, 0.4 to 0.7 as a moderate correlation, and 0.8 to 1 as a strong correlation were adopted. A difference was considered statistically significant when the *p*-value was less than 0.05.

Bioestat version 5.3 software (Mamirauá Institute, Tefe, AM, BRA) was used for the statistical analysis.

## Results

Thirty-four collections were performed. Other collections were not performed because it was clinically impossible or due to logistics at hospital discharge. Nine collections were disregarded due to the problem of non-controllable noise in the environment or agitation of the preterm infant, generating unreliable data.

The final sample consisted of 25 samples, 15 (60%) at moment I and 10 (40%) at moment II, expressed as means and standard deviations. The mean difference between HRV collection at moment I and moment II was 32.8 days. The data sets supporting the findings are included in an additional article archive (Additional file 1).

Table 1 shows the values obtained for the linear indices of the HRV in the frequency domain (SDNN, rMSSD, and pNN50) and non-linear indices (SD1, SD2, SD1/SD2, ApEn, and SampEn) at moment I and moment II. The study revealed statistically significant differences in the SD1, SD1/SD2, and SampEn indices.

**Table 1:** Values of HRV of premature infants related to length of hospital stay

	Moment I	Moment II	*p* value
HR (bpm)	154,6 (154) ± 7,01	162 (162) ± 12,44	0.0694
SDNN (ms)	15.14 (12.60) ± 9.87	12.38 (9.10) ± 9.01	0.170^a^
rMSSD (ms)	5.38 (3.60) ± 3.32	11.42 (6.60) ± 12.02	0.135^a^
pNN50 (%)	0.22 (0) ± 0.31	2.19 (0.30) ± 4.77	0.170^a^
SD1 (ms)	1.28 (0.60) ± 1.79	8.06 (4.70) ± 8.51	0.0007^a*^
SD2 (ms)	21.80 (18.50) ± 13.76	27.26 (24.00) ± 16.68	0.166^a^
SD1/SD2	7.55 (7) ± 4.74	2.70 (2.22) ± 1.73	0.003^*^
ApEn	0.87 (0.81) ± 0.46	1.03 (1.12) ± 0.44	0.12^a^
SampEn	0.81 (0.70) ± 0.33	1.07 (1.14) ± 0.23	0.0069^*^

*HR* heart rate, *bpm* Beats per minute, *ms* milliseconds, *%* percentage, *HR* heart rate, *SDNN* standard deviation of the mean of all normal RR intervals, *rMSSD* square root mean squared differences between adjacent normal RR intervals, *pNN50* percentage of pairs of consecutive RR intervals whose difference is greater than or equal to 50 m, *SD1* standard deviation of instantaneous beat-to-beat variability, *SD2* standard deviation of long-term continuous RR intervals, *SD1 / SD2* ratio between SD1 and SD2, *ApEn* approximate entropy, *SampEn* entropy of the sample

^a^Mann-Whitney

*p < 0.05

To analyse the influence of preterm infant age on the HRV indices, correlation of the data presented in Table 2 was performed. A negative and moderate correlation was observed in SD1/SD2, and a positive and moderate correlation was observed in ApEn and SampEn. These results were all statistically significant.

**Table 2:** Correlation between the age of the preterm infants and variables of the HRV analysis

	R (Pearson)	P (Pearson)	R (Spearman)	P (Spearman)
SDNN (ms)	–	–	0.31	0.12
rMSSD (ms)	–	–	−0.01	0.96
pNN50 (%)	–	–	0.34	0.09
SD1 (ms)	–	–	0.37	0.06
SD2 (ms)	–	–	−0.04	0.81
SD1/SD2	–	–	−0.47	0.01*
ApEn	0.43	0.03*	–	–
SampEn	0.46	0.02*	–	–

*ms* milliseconds, *%* percentage, *SDNN* standard deviation of the mean of all normal RR intervals, *rMSSD* square root mean squared differences between adjacent normal RR intervals, *pNN50* percentage of pairs of consecutive RR intervals whose difference is greater than or equal to 50 m, *SD1* standard deviation of instantaneous beat-to-beat variability, *SD2* standard deviation of long-term continuous RR intervals, *SD1 / SD2* ratio between SD1 and SD2, *ApEn* approximate entropy, *SampEn* entropy of the sample

*p < 0.05

**Fig. 1:**
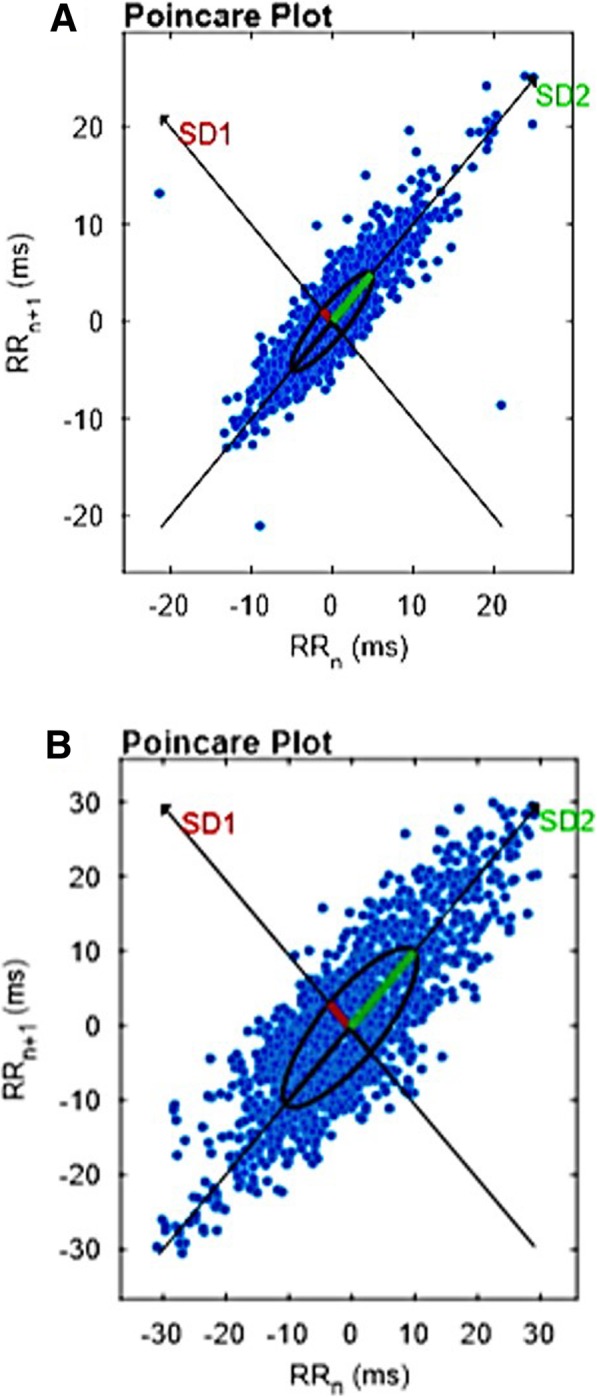
Illustration of the graphical analysis of the preterm infants in the Poincare Plot. **a** moment I, lower complexity of the indices (known as “torpedo”). **b** moment II, more dispersed patterns are related to greater complexity of the indices (known as “comet”)

A form of the analysis that illustrates the results obtained in the non-linear analysis of HRV and the graphical analysis from the Poincaré plot, where narrower patterns are related to a lower complexity of the indices (known as “torpedo”) and more dispersed patterns are related to a greater complexity of the indices (known as “comet”), are shown in Fig. 1.

## Discussion

The neurological development of preterm infants is limited due to prematurity and the cumulative effect of health management [5, 15]. Thus, it was relevant when there was an increase in PNS activity and HRV, and these results were obtained in our study.

At moment I, the values obtained in the HRV in the non-linear analysis were less complex, presenting a moderate correlation with greater age in preterm infants. This is compared with the number of occurrences, which also occurred with the increase of the SNS and PNS modulation demonstrated by the linear analysis of the time domain [5, 16]. These data suggest that the system is not yet fully mature in preterm infants [17].

A study enrolling newborn preterm infants found that HRV is progressively present in the first 3 to 4 days of life and forms a stable base after this period; the study further demonstrated that there is a reduction in ANS modulation at the onset of preterm infant life [18]. With the inadequate functioning of the SNA, the organism tends towards linearity, with the expected changes and adaptations not occurring. In this sense, it is relevant to observe the clinical evolution of preterm infants through HRV [19, 20]. In this study, the age of preterm infants presented a moderate and positive correlation in the ApEn and SampEn indices and moderate and negative correlation with the SD1/SD2 index, which were statistically significant, their relation to the sympathetic and parasympathetic systems need further clarification, increased HRV complexity and in this population can be considered a maturation of ANS.

In our study, there was no difference in the HR values between moments I and II, which shows the importance of including the HRV analysis in this population, where we found an increase in the HRV index at time II, which was statistically significant in SD1 and SD1/SD2. These measures relate to a key variable, the mean RR intervals, which allows the comparison of HR fluctuations [5].

The results characterized an increase in the pattern complexity (rMSSD, pNN50, and SD2) and PNS (rMSSD, pNN50, SD1, and SD2), highlighting the Poincaré plan markers (SD1, SD2, SD1/SD2, and ApEn) and HRV, which demonstrated increased HRV complexity during hospitalization with an emphasis on the evolution of cognitive and progressive development for eutrophy [5–7, 21].

The data obtained at moment II have a clinical characteristic favourable to this population, although discussion of the subject is limited in this paper. These data are not addressed in the study of autonomy in preterm infants, a subject that has been under discussion thus far [22, 23].

The use of HRV in preterm infants is limited in its use and analysis because of the noisy environment in the preterm infant clinic and during hospitalization related to the stress of the hospital, which can generate physiological changes in neonates [24–26]. This variable was not used in this study and served only as an exclusion criterion when noise interfered with the quality of data collection.

The findings of this study contribute to the inclusion of the use of HRV as a complementary tool to evaluate the maturation of the ANS in preterm infants. It is an easy and quick application already recommended by other authors for this population as a biomarker for the clinical evaluation of pain, stress, sepsis, necrotizing enterocolitis, and intraventricular haemorrhage [13, 27–31]. However, to include HRV in the evaluation of the clinical course of preterm infants, it is necessary to determine its behaviour [18].

### Study limitations

As limitations of this study, we can highlight the restricted sample size and absence of analysis of other variables that may be related to the clinical evolution and ANS in preterm infants, such as the presence of neurological lesions or the occurrence of low weight and malnutrition [28, 32]. As an additional limitation, we see the lack of long-term follow-up, is the lack of published data with which to compare our results.

## Conclusion

The data demonstrate an increase in HRV values ​​in preterm infants at moment II, demonstrating development in SNA maturation and increased SNS activity, PNS, and HRV complexity during hospitalization. HRV use may be a useful tool to evaluate clinical evolution in this population.

From this study, we suggest the use of HRV as a supplementary tool to evaluate preterm ANS during hospitalization to assist in the verification of its clinical evolution. We also suggest new studies with larger sample sizes and clinical evaluation times to determine the behaviour of HRV in this population and to carry out an approach that makes it possible to correlate HRV with other clinical characteristics of the development of preterm infants, as well as the performance of multicentre studies.

## Additional file

Additional file 1: HRV premature database. (XLSX 13 kb)

## Data Availability

The dataset supporting the conclusions of this article is included within the article (Additional file 1).

## Abbreviations

ANS: Autonomic nervous system
ApEn: Approximate entropy
HR: Heart rate
HRV: Heart rate variability
pNN50: Percentage of adjacent RR intervals with duration difference greater than 50 ms
PNS: Parasympathetic nervous system
rMSSD: Square root mean square differences between adjacent normal RR intervals in one time interval
SampEn: Sample entropy
SD1: Dispersion of points perpendicular to the line of identity that appears to be an index of instantaneous recording of beat-to-beat variability
SD1/SD2: Ratio between short and long variances of RR intervals
SD2: Points scatter along the identity line, which represents the HRV in a long-term record
SDNN: Standard deviation of all normal RR intervals
SNS: Sympathetic nervous system
